# Reduction in the metabolic levels due to phenotypic plasticity in the Pyrenean newt, *Calotriton asper*, during cave colonization

**DOI:** 10.1002/ece3.6882

**Published:** 2020-10-12

**Authors:** Olivier Guillaume, Marine Deluen, Allan Raffard, Olivier Calvez, Audrey Trochet

**Affiliations:** ^1^ Station d'Ecologie Théorique et Expérimentale UMR 5321 CNRS Université Paul Sabatier Moulis France

**Keywords:** adaptation, *Calotriton asper*, cave colonization, metabolic rate, phenotypic plasticity

## Abstract

According to theories on cave adaptation, cave organisms are expected to develop a lower metabolic rate compared to surface organisms as an adaptation to food scarcity in the subterranean environments. To test this hypothesis, we compared the oxygen consumption rates of the surface and subterranean populations of a surface‐dwelling species, the newt *Calotriton asper,* occasionally found in caves. In this study, we designed a new experimental setup in which animals with free movement were monitored for several days in a respirometer. First, we measured the metabolic rates of individuals from the surface and subterranean populations, both maintained for eight years in captivity in a natural cave. We then tested individuals from these populations immediately after they were caught and one year later while being maintained in the cave. We found that the surface individuals that acclimated to the cave significantly reduced their oxygen consumption, whereas individuals from the subterranean population maintained in the cave under a light/dark cycle did not significantly modify their metabolic rates. Second, we compared these metabolic rates to those of an obligate subterranean salamander (*Proteus anguinus*), a surface aquatic Urodel (*Ambystoma mexicanum*), and a fish species (*Gobio occitaniae*) as references for surface organisms from different phyla. As predicted, we found differences between the subterranean and surface species, and the metabolic rates of surface and subterranean *C. asper* populations were between those of the obligate subterranean and surface species. These results suggest that the plasticity of the metabolism observed in surface *C. asper* was neither directly due to food availability in our experiments nor the light/dark conditions, but due to static temperatures. Moreover, we suggest that this adjustment of the metabolic level at a temperature close to the thermal optimum may further allow individual species to cope with the food limitations of the subterranean environment.

## Graphical abstract

We showed evidence of a phenotypic plasticity at the metabolic level as a mechanism of cave colonization and adaptation in a european newt.

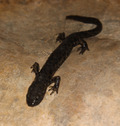

## 1 INTRODUCTION

Under climatic changes, some surface organisms use subterranean environments where temperatures are buffered over time. Although global warming currently affects the subterranean ecosystems, extreme temperatures are softened. When these extremes constitute a physiological challenge for species at the surface, the subterranean habitats may serve as refugia for some of them (Mammola et al., 2019). Since climate changes are also buffered over geological time, some organisms may have exploited these environments and persisted, while the original surface range became uninhabitable (Humphreys, 2000). However, subterranean ecosystems are considered to be depauperate in terms of both biodiversity and biomass (Romero, 2009), mainly because the food supply is a strong limiting factor (Hüppop, 2000). However, without solar radiation, photosynthesis, or primary producers, the basic food resource in most caves is the organic matter from external sources. Thus, except for some subterranean habitats under the tropics, food scarcity exists in the subterranean habitats, which may be a tremendous selective pressure for organisms (Juberthie & Decu, 1994; Hüppop, 2000; Poulson, 2001; Culver and Pipan, 2009; Romero, 2009). Hence, most subterranean species find their food outside the cave, while few species exploit the poor local resources. To cope with this limitation, theories on cave adaptation suggest that several characteristic features have evolved in cave dwellers, such as an increase in the foraging ability, starvation resistance, and a reduction in energy demand (Hervant & Renault, 2002). As part of a strategy of energy optimization, a low basal metabolic rate has been observed in many cave species (Hüppop, 2000).

The basal or standard metabolic rate reflects the minimum amount of energy required to maintain body processes (Norin & Metcalfe, 2019). Metabolic rate can strongly vary among and within species, even after categorizing the species based for instance on size, sex, and age. Additionally, metabolic rate demonstrates plasticity and varies in response to changing environments. In particular, metabolic rate can be highly plastic across time within individuals, owing to seasonal changes as well as among individuals (i.e., intrapopulation differences in energy demands) and among populations, owing to local adaptation (Fox et al., 2019). Phenotypic plasticity (i.e., the capacity of a single genotype to exhibit variable phenotypes in different environments) has long been recognized to provide many ecological benefits and is often highly adaptive. It allows for the rapid development of a better phenotype–environment match, and in many systems, the costs of phenotype and limits to plasticity outweigh the costs of plasticity (DeWitt et al., 1998; Murren et al., 2015).

However, few studies have focused on plasticity in cave environments. To date, most studies have compared metabolic rates between closely related subterranean and surface species, or within cave species, to estimate their adaptation level (Mejía‐Ortíz & López‐Mejía, 2005). Due to the phylogenetic distance, such comparative studies are often limited in their conclusion, and the involved processes remain controversial (Culver and Pipan, 2009; Poulson, 2011; Romero, 2009). Issartel et al. (2010) reported that the colonization of cave environment by the Pyrenean newt, *Calotriton asper* (which mainly live on the surface) implied a fasting adaptation with acquisition of a lower basal metabolic rate. A local adaptation cannot be ruled out, as cave‐dwelling populations have been genetically isolated from the surface populations (Mila et al., 2010; Valbuena‐Ureña et al., 2018). However, recent studies in the fish *Astyanax mexicanus* suggested that changes accompanying cave colonization can be established rapidly at the morphological and behavioral levels (McGaugh et al., 2019) and that several cave‐related traits appear within a single generation due to phenotypic plasticity (Bilandžija et al., 2020).

Consequently, we investigated whether acclimation to cave conditions may induce plasticity of metabolic rates on the surface *of C. asper*. To do so, we compared the metabolic rate of surface *C. asper,* before and after acclimation to cave life, to the metabolic rate of a subterranean population of C. *asper*. We expected a lower metabolic rate of surface *C. asper* acclimated to the cave than that of subterranean *C. asper*. Basal metabolic rate is usually assessed indirectly as the rate of oxygen consumption using standard techniques and conditions, including resting during the inactive circadian phase (White & Kearney, 2013). These conditions are difficult to control in cave organisms, considering that activity can be conserved, relaxed, or lost depending on the cave species (Friedrich, 2013). Another classical approach consists of quantifying the costs of animal activities, which form the major part of energy expenditure by animals above resting metabolic rate. In addition, a number of proxies for energy expenditure in free‐living animals have been proposed, such as heart rate and dynamic body acceleration, although assessing these proxies is still challenging depending on the species (Wilson, 2020). This assessment is not easy in cave Urodel, whose scores are expected to be weak, and can badly react to experimental devices along with a response such as apathy or overactivity (Chin et al., 2018; Guillaume, 2000). Since stress has a great impact on the metabolic level (Rabasa & Dickson, 2016), we conceived a new experimental setup to limit this reaction and measure oxygen consumption continuously as a proxy for several days in free‐moving animals. To validate our experimental setup and provide a framework for comparison of the potential plasticity of the metabolic rate of *C. asper*, we monitored the rate of a cave‐dwelling population of *C. asper*, the troglobitic (i.e., obligate or strictly subterranean animals), aquatic Urodel *Proteus anguinus,* and two surface aquatic species: the Urodel *Ambystoma mexicanum*, which is a neotenic form and a reared species frequently used in research as a good Urodela taxon reference, and the benthic fish *Gobio occitaniae*, which is common in the clear water systems of southwestern France. We expect the *C. asper* metabolic rate to lie between those of the strictly subterranean and surface species.

## 2 MATERIALS AND METHODS

### 2.1 Study species

The Pyrenean newt *Calotriton asper* (Photograph 1) is a brook newt, endemic to the French, Andorran, and Spanish Pyrenees. It lives mainly in rivers and lakes at altitudes between 400 and 2,500 m; however, several cave populations have been found (Clergue‐Gazeau & Martinez‐Rica, 1978; Mila et al., 2010). Although lungs are absent in the adult phase, the Pyrenean newt *Calotriton asper* is entirely aquatic (Clergue‐Gazeau, 1971).

The proteus, *Proteus anguinus* (Photograph 2), is the only obligate cave‐adapted vertebrate in Europe, exhibiting troglomorphic characteristics, such as skin depigmentation, eye degeneration, and neoteny (Aljančič, 2019; Bulog, 1994).

The axolotl, *Ambystoma mexicanum,* is a neotenic Urodel that is widespread in the laboratories and aquariums throughout the world but is endangered in the wild (Bride et al., 2008).

The gudgeon *Gobio occitaniae* is a benthic fish locally present in lotic clear water systems in southwestern France (Keith et al., 2011).

### 2.2 Experimental setup

Twenty individuals from each species (and populations) were tested for each treatment (constant dark or 12:12 (day:night) cycle; except *P. anguinus*, which was exposed to light, *N* = 8). The individuals were isolated in a 10 L transparent plastic chamber filled with water from the cave river (10.13 ± 0.19 ppm O_2_, 9.90 ± 0.21°C), where they had free movement. The oxygen concentration and the temperature in the tank were monitored using a FireSting fiber‐optical oxygen meter (PyroScience company). During a three‐day acclimatization period, the individuals were exposed to the treatment (constant dark or 12:12 (day:night) cycle). The water was continuously renewed during this period. At the start of the measurement period, the flow was discontinued and the oxygen concentration and temperature of each chamber was monitored every 30 min. The tests were terminated when the oxygen concentration decreased to 4 ppm to avoid physiological stress.

### 2.3 Animal origin and maintenance

Surface *C. asper* adults were captured from Cailla Brook (600–1,000 m, longitude: 42.805993°, latitude: 2.190989°). The first group was caught in November 2006 and the second in October 2015 (i.e., after the breeding season). They were maintained in the cave of the “Station d'Ecologie Théorique and Experimentale, i.e., SETE” (Ariège, France). The first group was kept under total darkness for eight years before metabolic measurement. The second group was exposed to alternating 12 hr day/night photic cycle for one year. The first series of metabolic measurements were performed a few days after the capture and the second series after 1 year. To mimic the conditions of the day, we used full spectrum daylight fluorescent lamps (True‐light ®Natural Daylight 5500K/ Color Rendering 1 A, Ra > 90).

Subterranean *C. asper* adults were captured in the cave of Labouiche (43.01231°, 1.342365°). The first group was caught in November 2006 and the second in November 2015. They were maintained in the cave of the SETE. The first group was kept in total darkness for eight years. Metabolic measurement of the second group was performed three times: (a) few days after the capture in total darkness, (b) then, few days after, under alternating 12 hr day/night cycles, (c) and after one year under alternating 12 hr day/night cycles.

*Proteus anguinus* and *A. mexicanum* could not be captured from the wild, and therefore, we used individuals from the breedings. *Proteus anguinus* species were bred and maintained for more than 20 years in the cave of SETE in total darkness. The first metabolic measurement was performed in darkness on 20 individuals. Then, eight of them were randomly chosen and exposed to the 12 hr day/night cycle (from the three‐day acclimatization period), and the metabolic rates were then measured. Lastly, these individuals were exposed to the 12 hr day/night cycle in the cave for one year before being tested again.

Adult *A. mexicanum* was supplied by a private breeder. They were maintained in the cave of SETE and exposed to 12 hr day/night conditions for several months before their metabolic rates were measured.

Adult *Gobio occitaniae* was fished from the Vère in Cahuzac (43.984917°, 1.911782°). They were kept under 12 hr day/night in the cave of SETE, and their metabolic rates were measured after 10 days.

When outside the measurement period, the animals used in this study were fed ad libitum with live chironomid larvae once a week.

### 2.4 Statistical analysis

The variations in oxygen concentrations over time were analyzed using linear regression tests (XLstat software). Oxygen consumption was assessed as the slope of the regression between time and oxygen concentration in the tank divided by the body mass of the tested individuals (Oxygen consumption = slope of time and oxygen∕body mass of the tested individuals). The difference in oxygen consumption levels between species, populations, or treatments among the same species were analyzed using the Steel–Dwass–Critchlow–Fligner bilateral test for multiple comparisons of pairs (XLstat software). The differences in the oxygen consumption level between surface *C. asper* just caught and after 1 year in the cave were analyzed using the Wilcoxon unilateral test (XLstat software). The differences in the consumption level exhibited by individuals (subterranean *C. asper* or *P*. *anguinus*) in darkness, exposed to the day/night cycle, and after 1 year under the day/night cycle were analyzed using the Friedman test (XLstat software).

## 3 RESULTS

**Figure 1:**
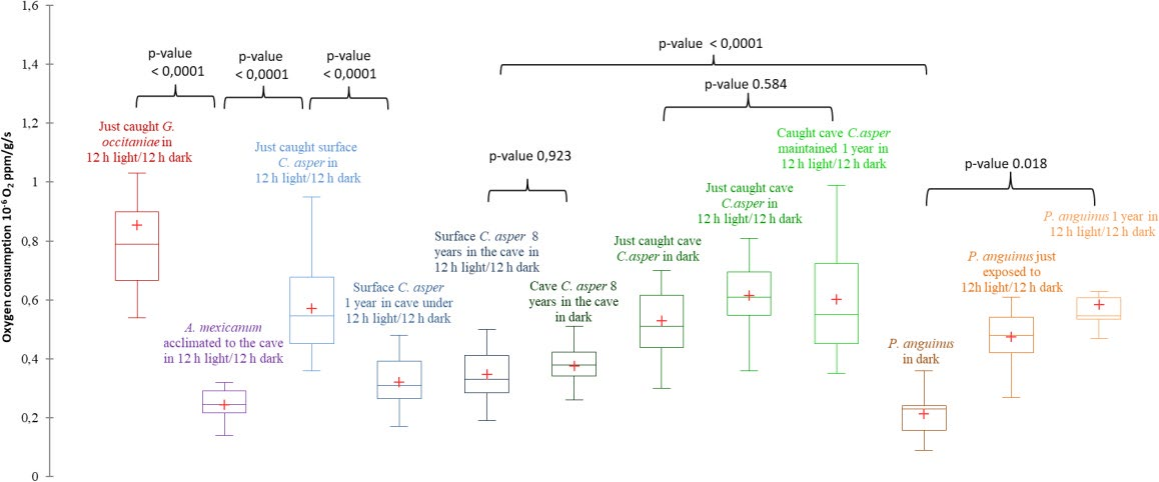
Comparison of the oxygen consumption level in ppm per gram of body mass per second for *Gobio occitaniae* caught and tested in the cave under the 12 hr light/dark cycle (*N* = 20, red); reared *Ambystoma mexicanum* for several months in the Moulis cave under the 12 hr light/dark cycle (*N* = 20, violet); surface *Calotriton asper* just caught and exposed in the Moulis cave to the 12 hr light/dark cycle (*N* = 20, light blue); the same surface *C. asper* after one year under the 12 hr light/dark cycle in the cave (*N* = 20, medium blue); surface *C. asper* maintained for eight years in the cave under the 12 hr light/dark cycle (*N* = 20, dark blue); cave *C. asper* maintained for eight years in the cave in dark (*N* = 20, dark green); cave *C. asper* just caught and tested in the Moulis cave in dark (*N* = 20, olive green); the same cave *C. asper* after the 12 hr light/dark cycle (*N* = 20, lime green); the same cave *C. asper* after one year in the cave under the 12 hr light/dark cycle (*N* = 20, light green); *P. anguinus* reared in the Moulis cave for several years in dark (*N* = 20, dark orange); the same *P. anguinus* tested under the 12 hr light/dark cycle (*N* = 8, medium orange); and the same *P. anguinus* tested 1 year after the 12 hr light/dark (*N* = 8, light orange). The red crosses correspond to the means. The central horizontal bars are the medians. The lower and upper limits of the box are the first and third quartiles, respectively. The minimum is shown at the far bottom of the whisker, and the maximum at the far top

Oxygen concentrations for all individuals decreased linearly over time (Table S1). Since we selected a lower ppm threshold to terminate the test, the metabolic rate was monitored for about 6 to 15 days (Table S2). Surface *C. asper* exhibited higher metabolic rates than subterranean *C. asper* (Figure 1). However, the rates of individuals from the same surface population after 8 years in the cave in darkness were reduced to levels close to that of the subterranean individuals.

Moreover, when individuals from the surface population of *C. asper* were maintained in the cave under the day/night photic cycle for one year, their oxygen consumption significantly decreased (Figure 1). We did not find any significant changes in individuals from the subterranean population exposed to the day/night cycle even after one year. In contrast, exposure to the photic cycle increased the rate of *P. anguinus,* and the difference became significant after one year.

The oxygen consumption rate of cave *P. anguinus* was lower than that of other species; however, the difference was not significant compared to that of epigean *A. mexicanum*. On the contrary, the oxygen consumption rates of *G. occitaniae* few days after their capture were consistently higher than those of the urodelan species.

## 4 DISCUSSION

Variation in the metabolic rate between species is mostly explained by phylogeny, body mass, and a range of environmental factors, namely food availability and temperature (Burton et al., 2011). In the subterranean organisms, a reduction in the metabolic rate is considered by many authors as an adaptative character to cave life that can be achieved in different ways, such as a reduction in body size as well as physiological depression (Poulson, 1971; Culver, 1982; Hüppop, 2000; Passow et al., 2015). In this study, we controlled the potential effects of body mass, food supply, and temperature and found among‐species variation, effects of the environment due to light/dark exposure, and the duration of the maintenance in cave conditions. For instance, the gudgeons showed a higher metabolic rate. Many factors can explain this score, including the high phylogenetic distance with other species from the Urodela taxon or the lifestyle of gudgeons, which is a more active species. Yet the difference was not significant between *P. anguinus* and the epigean, *A. mexicanum*. The ability of *A. mexicanum* to acclimatize to thermal conditions has long been documented (Whitford & Sherman, 1968; Irwin et al., 1998). Although experiments on this salamander were not previously conducted at low temperatures of approximately 10°C, we suspect a passive effect of cooling during long‐term maintenance under cold conditions in caves, considering that this salamander is tolerant to very cold conditions (Scott, 1996). In brief, our new experimental setup allowed us to analyze the oxygen consumption rate of benthic subterranean and surface aquatic Urodela with continuous measurements during free movement for several days. We thus assume that (a) this method is applicable for few active organisms with low oxygen consumption when standard techniques and conditions or proxies for energy expenditure are challenging to implement and (b) the measured oxygen consumption rates are good approximations of the routine metabolic activity with a realistic degree relatively close to the natural conditions.

Among the varying environmental factors in this study, the light/dark regime has disparate effects on species. We found that the effect of light was immediate in the strictly subterranean species, *P. anguinus*. The metabolic rate increases, probably due to stress, as animals avoid light (Durand, 1998; Guillaume, 2000), and production of melanin, as individuals show pigmentation after several weeks. On the contrary, light had no effect on the metabolic level of *C. asper*, perhaps because it is both a diurnal and nocturnal creature. Therefore, the reduction in the metabolic levels of cave‐acclimated *C. asper* can be more likely ascribed to the rapid acclimatization to the cave temperature.

The time frame for the evolution of traits in cave animals is poorly documented; however, there is evidence that the fish species *Astyanax mexicanus*, can rapidly evolve due to changes in the genotypic and phenotypic levels, particularly by phenotypic plasticity (McGaugh et al., 2019; Bilandzija et al., 2020). Our results on *C. asper* demonstrated plasticity at the metabolic level during acclimatization to the cave. However, we investigated the advantages of colonization of this habitat by *C. asper*. The thermal optimum range for the development of both subterranean and surface *C. asper* is 12–15°C, which is slightly above the temperature range observed in caves (Clergue‐Gazeau, 1971; Guillaume, 2000). Similarly, for *P. anguinus*, whose thermal optimum is 16°C, temperature never reached in its natural habitats (Durand, 1998). Thus, the shift in the metabolic level observed in the acclimated *C. asper* might be due to the static temperatures within a favorable range close to the thermal optimum. This hypothesis requires further investigation, taking into account the potential effects of fluctuating temperatures. However, a study on the Arctic charr *Salvelinus alpinus* demonstrated that compared to constant temperatures, even small temperature fluctuations could increase the routine rates of oxygen consumption (Lyytikäinen & Jobling, 1998). Moreover, there is evidence that *C. asper* utilizes behavioral adjustments to maintain the body temperature in their specific temperature range and select temperatures in order to compensate for low opportunities of favorable temperatures through contrasting seasonal climatic variations (Trochet et al., 2018). The static conditions of the underground climate compared to the surface offers the stenothermal ectotherms, such as *C. asper,* certain conditions that are close to homeothermy near its thermal optimum. Thus, taking refuge into caves may be an efficient alternative strategy to face seasonally changing environments. Under such climate changes, various external organisms may be able to exploit subterranean environments as refugia (Mammola et al., 2019). For instance, movement of faunas from surface to subterranean habitats, and vice versa, have been reported in athropods, particularly in the mountainous areas, under adverse surface climate and habitat changes (Ledesma, 2020).

In addition, some recent studies suggest that *C. asper* probably recolonized the Pyrenees from distinct glacial refugia (Lucati et al., 2020). Under these conditions, few populations may have colonized caves to escape from the climatic changes occurring outside (Guillaume, 2001; Miaud & Guillaume, 2005). Based on our study, these events could have been favored through plasticity of the metabolic rate due to an adjustment of the temperatures in refugia such as caves, which may further allow individuals to cope with food limitations in such environments. Indeed, the physiological traits in animals, although short‐term, have long‐term causes driven by evolutionary processes, thereby indicating that initial advantages could lead to further cumulative advantages (Fox et al., 2019).

## 5 ANIMAL ETHICS

All the required French permits for animal experimentation on the species used in this study were obtained from the Animal Experimentation Accreditation: n°A09583 for the lab, n° A09‐1 (2001)‐A09‐2 (2007)‐A09‐3(2011) ‐ A09‐5(2013) for the experimenters, and n° 09–19, 09–273, 09–295 for the animal caretakers and handlers to use wildlife for scientific purposes.

## 6 FIELD STUDY PERMISSIONS

All the required French permits (permit no. 2017–04, 2007–11–1342, and 2016‐s‐01) for authorization of capture, marking, transport, detention, use, and release of protected amphibian species, along with animal experimentation permits were obtained, and the project was approved by the National Council for Nature Protection on March 19, 2007, and the Regional Scientific Council for the Natural Heritage of the Region Languedoc‐Roussillon‐Midi‐Pyrénées on April 5, 2016.

## CONFLICT OF INTERESTS

The authors declare that they have no conflict of interest.

## AUTHOR CONTRIBUTION

**Olivier Guillaume:** Conceptualization (lead); Data curation (lead); Formal analysis (lead); Funding acquisition (lead); Investigation (lead); Methodology (lead); Project administration (lead); Resources (equal); Software (lead); Supervision (lead); Validation (lead); Visualization (lead); Writing‐original draft (lead); Writing‐review & editing (lead). **Marine DELUEN:** Investigation (supporting); Methodology (supporting); Resources (supporting); Writing‐original draft (supporting). **Allan Raffard:** Formal analysis (supporting); Investigation (supporting); Writing‐original draft (supporting). **Olivier Calvez:** Investigation (supporting); Project administration (supporting); Writing‐original draft (supporting). **Audrey Trochet:** Methodology (supporting); Writing‐original draft (supporting).

## Supporting information

Table S1‐S2

## Data Availability

Data are deposited in the Dryad public repository (https://doi.org/10.5061/dryad.905qfttj6)
