# Supplementary material for: Reduction in the metabolic levels due to phenotypic plasticity in the Pyrenean newt, Calotriton asper, during cave colonization
Source: Ecol Evol. 2020 Oct 12;10(23):12983–9. doi: 10.1002/ece3.6882 (PMC7713917; doi:10.1002/ece3.6882)
Supplement: Supplementary file 1 — Table S1‐S2 [file ECE3-10-12983-s001.docx]

**Supplementary material**

**Tables**

Table 1. Linear regression analysis (R² coefficient of determination, Fisher's F test and P-value) of the oxygen consumption per gram of body mass over time for twenty individuals from various species. *C. asper*, *Calotriton asper; P. anguinus*, *Proteus anguinus; A. mexicanum,* *Ambystoma mexicanum; G. occitaniae, Gobio occitaniae.*

| Species | ***C. asper*** | | | | ***P. anguinus*** | ***A. mexicanum*** | ***G. occitaniae*** |
| --- | --- | --- | --- | --- | --- | --- | --- |
|  | **Epigean** | | **Hypogean** | |  |  |  |
|  | Just caught | Acclimated  to the cave | Just caught | Acclimated to the cave |  |  |  |
| Average duration of tests in days | 14.4 ± 1.3 | 15.3 ± 1.7 | 15.5 ± 1.8 | 15.5 ± 1.9 | 15.3 ± 0.9 | 8.4 ± 1.3 | 6.0 ± 0.6 |
| Average mass in grams | 8.4 ± 1.6 | 9.5 ± 1.7 | 7.4 ± 2.1 | 7.2 ± 2.3 | 11.8 ± 3.7 | 32.4 ± 5.9 | 14.5 ± 6.1 |

Table 2. Average (± SE) duration of tests and body mass of tested individuals (N = 20). *C. asper*, *Calotriton asper*; *P. anguinus*, *Proteus anguinus; A. mexicanum,* *Ambystoma mexicanum; G. occitaniae, Gobio occitaniae*
